# Can Aggregate-Associated Organisms Influence the Fouling in a SWRO Desalination Plant?

**DOI:** 10.3390/microorganisms10040682

**Published:** 2022-03-22

**Authors:** Tamar Jamieson, Harriet Whiley, Jason R. Gascooke, Sophie C. Leterme

**Affiliations:** 1 College of Science and Engineering, Flinders University, P.O. Box 2100, Adelaide, SA 5001, Australia; harriet.whiley@flinders.edu.au (H.W.); jason.gascooke@flinders.edu.au (J.R.G.); sophie.leterme@flinders.edu.au (S.C.L.); 2 Flinders Institute for NanoScale Science and Technology, Flinders University, P.O. Box 2100, Adelaide, SA 5001, Australia

**Keywords:** biofouling, biofilm, seawater reverse osmosis, marine snow, aggregate, TEP

## Abstract

This pilot study investigates the formation of aggregates within a desalination plant, before and after pre-treatment, as well as their potential impact on fouling. The objective is to provide an understanding of the biofouling potential of the feed water within a seawater reverse osmosis (SWRO) desalination plant, due to the limited removal of fouling precursors. The 16S and 18S rRNA was extracted from the water samples, and the aggregates and sequenced. Pre-treatment systems, within the plant remove < 5 µm precursors and organisms; however, smaller size particles progress through the plant, allowing for the formation of aggregates. These become hot spots for microbes, due to their nutrient gradients, facilitating the formation of niche environments, supporting the proliferation of those organisms. Aggregate-associated organisms are consistent with those identified on fouled SWRO membranes. This study examines, for the first time, the factors supporting the formation of aggregates within a desalination system, as well as their microbial communities and biofouling potential.

## 1. Introduction

Oceanic microorganisms can secrete a diverse array of large molecules, collectively called extracellular polymeric substances (EPS) [1]. While EPS are believed to be the precursors of biofilm formation, in open-water environments, they contribute to the formation of organic colloids and larger aggregations of cells, called particulate organic matter (POM) or ‘marine snow’. POM, a source of carbon and nutrients to heterotrophic microorganisms, is essential for the transport of elements and energy towards the deep ocean and the main method for the removal of carbon from surface waters [2,3]. POMs harbour a diverse and complex disparity of inorganic particles and can be regarded as microhabitats, due to the large amount of autotrophic and heterotrophic organisms found within [3]. POM’s microbial community abundances can reach up to two orders of magnitude higher than the surrounding seawater environment [4]. The high microbial activity of POM-associated (PA) bacteria is reflected by their enhanced cell-specific rates of polymer hydrolysis and substrate uptake, relative to the free-living (FL) bacteria in the surrounding water [5]. In their studies, Milici et al. [6] showed remarkable taxonomic differences between PA and FL bacteria in the deep Southern Ocean water masses. PA-bacterial communities had high numbers of polymer-degrading bacteria, such as Flavobacteria, γ-proteobacteria, Planctomycetes, and Verrucomicrobia, whereas the FL bacterial communities had high numbers of α-protebacteria [7,8,9]. The PA communities are commonly found in marine biofilms, especially as biofilm initiators [10,11,12,13,14]. In particular, γ-proteobacteria perform an important role within marine biofilms, especially through their capability for polysaccharide biodegradation and cellulose metabolism [10,15,16]. It can also dominate the initial phase of biofilm formation (Rampadarath et al., 2017). However, the FL community of α-proteobacteria have also been known to dominate all stages of biofilms [13,14,17]. 

Much like the colonization of the surfaces, the colonization of aggregates by bacteria is complex and occurs in several steps. First, bacteria will attach loosely to the aggregate. This attachment will gradually increase, until the cells are permanently attached; then, growth rates of the attached bacteria will drive the colonization over attachment [18]. Fast moving bacteria will encounter an aggregate in about <1 day [19], and non-motile bacteria will collide with aggregates at a lower frequency, due to the motion of the liquid they are in. Eventually, the total number of cells on the aggregate will increase, and the bacterial community becomes established, much like during the formation of bacteria biofilms on inert surfaces. Biofilm formation is an impediment for many water treatment infrastructures, such as desalination plants, as membrane biofouling is considered to be a major contributor to the increase in production costs [20]. Biofouling of the SWRO membrane is often described as the accumulation of complex sessile microbial communities, which are surrounded by an impenetrable, heterogeneous matrix of EPS, primarily comprised of polysaccharides and proteins [21]. To date, there has been limited research assessing the contribution of marine aggregates to the fouling of seawater reverse osmosis (SWRO) membranes in situ, and it is known that not all biofilm precursors can be removed by pre-treatment [22]. Recent studies have focused on the role of transparent exopolymer particles (TEP; marine snow pre-cursor) as potential precursors of SWRO membrane biofouling [23,24,25].

The limited removal of TEP from seawater via pre-treatments increases the biofouling potential [22]. Bar-Zeev et al. [26] proposed a new paradigm, stating that TEP plays a critical role alongside the “traditional” stages of biofilm formation and introduced the term “protobiofilm” to characterise TEP showing extensive microbial outgrowth and colonization. TEP are often found in marine environments and play a role in the formation and development of marine biofilms [21,27]. Within the desalination process, high levels of potential biofilm-forming TEP have been found to be reach the SWRO membrane [28]. Bar-Zeev, Passow, Romero-Vargas Castrillón, and Elimelech [27] highlight that a better understanding of TEP formation pathways, size spectrum, chemical nature, and bacteria interactions could instigate new pre-treatment methods for their efficient removal, as well as novel cleaning strategies following attachment to a membrane surface.

The production of fresh water via desalination has been extensively recognized as a valuable solution to ensure water security [29]. This is especially true in drought-affected areas and increasingly important, as global water shortages are predicted to be further exacerbated through climate change [30,31]. SWRO is a reliable and efficient process, enabling the separation of salts and water molecules through a semi-permeable membrane, due to a pressure and chemical potential gradient [29]. SWRO is considered the most suitable method for the production of potable water, as the increasing demand is greater than either groundwater or surface water treatment can supply [32]. Established biofilms, due to the complex nature of EPS, have been found to be impervious to oxidizing agents and biocides, making the extrication of biofilms problematic [33,34]. Pre-treatment systems are, thus, essential in SWRO facilities to moderate organic and inorganic fouling of the RO membranes. Multimedia filtration, as well as cartridge filtration, are frequently part of the coagulation/flocculation steps found in most pre-treatment systems [35,36]. The addition of ultrafiltration (UF) technology to the pre-treatment process of desalination plants has become more prevalent. A major advantage of UF technology is the ability to remove smaller size particles more effectively than multimedia filtration [37,38,39,40,41].

Previous studies have examined the fouling potential of feedwater, as well as the impact that it has on biofouling of SWRO membranes, both in laboratory settings and pilot-scale systems, with RO membrane biofouling monitored over time [42,43,44,45,46,47]. Other studies focused on the microbial communities of the cartridge filters and SWRO membranes, or on the validity of pre-treatment methods on the permeate communities’ post-treatment within desalination systems [45,48,49,50,51,52,53,54,55,56,57,58]. In this study, we explore the formation and composition of aggregates within a SWRO desalination system, pre- and post-treatment, and their influence in biofouling. Aggregates were formed in water collected from a SWRO desalination plant pre- and post-treatment. Comparison of the microbial composition of the aggregates was performed to ascertain the organisms associated with biofouling within the plant. Moreover, further comparisons were made, looking at the size and composition of the aggregates, in order to investigate how they could influence biofilm development on the SWRO membranes. The findings from this study will inform future strategies aimed at controlling the formation of aggregates to reduce membrane fouling.

## 2. Materials and Methods

### 2.1. Description of the Penneshaw SWRO Desalination Plant

**Figure 1:**
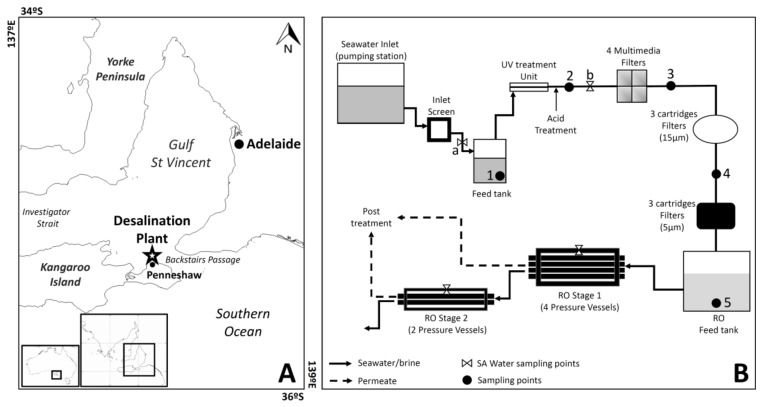
(**A**) Location of the Penneshaw SWRO desalination plant, and (**B**) schematic diagram of the Penneshaw SWRO desalination plant. Numbers indicate the different sampling points: (1) intake seawater, (2) post-MP-UV and acid treatment, (3) post-sand filter treatment, (4) post-cartridge filter treatment, and (5) SWRO feed tank water. The letters indicate the SA Water sampling points: (a) intake seawater and (b) intake seawater after acid treatment.

The Penneshaw SWRO desalination plant has a capacity of 300 kL·day^−1^ and has been described in detail in previous studies [59]. Seawater, from a depth of 6 m, is pumped from the coastal waters north of Kangaroo Island (South Australia) at a site located 200 m from the Penneshaw desalination plant (Figure 1) and enters the system through two pre-filtration screens (10 cm and 0.5 mm pore sizes, respectively). This is then followed by the pre-treatment system, which includes sulfuric acid addition, a medium pressure-ultraviolet (MP-UV) disinfection unit, four parallel multimedia filters (gravel, garnet, sand, and coal, with grain size ranging from 0.3 to 10 mm), and two consecutive sets of three-cartridge filters each, with pore sizes of 15 and 5 μm, respectively. The flow rate through the system is typically 8.4 L·s^−1^, after which the seawater enters the SWRO feed tank. Every 48 h, back wash occurs for each multimedia filters, at a flow of ~16 L/s for 420–510 s, and a forward rinse of raw seawater for 300 s at 5 L/s follows.

The Penneshaw SWRO unit is a single framework compartment comprising of 12 pressure vessels, each containing 4 membrane modules. The SWRO membrane modules are spiral-wound, thin-film composite of polyamide (FILMTEC^TM^ SW30HRLE-440i), with an active surface area of 41 m^2^. Four fouled membrane modules from the 1st and 2nd stage positions, after 2- and 4- years of service, were used in this study.

### 2.2. Water Sampling Sites

Seawater was collected at two sampling points within the desalination plant: (1) intake seawater, located prior to any treatment, and (2) pre-treated seawater, within the SWRO feed tank, located directly after the cartridge filters and before the SWRO membrane modules. Composite samples were collected in 2 L grabs, every 30 min, until a total volume of 20 L was collected. 

### 2.3. Formation of Aggregates

In this experiment, aggregates were produced in a 20 L clear carboy (Nalgene) using collected water. Microspheres (BioMag Carboxl; Bang Laboratories Inc., Fishers, IN, USA) were added to the carboy at a concentration of 2.5 × 10^5^ particles mL^−1^, following the protocols of Mari et al. [60]. 

#### 2.3.1. Visualisation of TEP in the Aggregates

Under vacuum, aggregates were filtered onto 0.4 μm polycarbonate filters (Merck Millipore Ltd., Darmstadt, Germany). The filters were then stained with alcian blue for 20 min before being washed with sterile seawater. Each filter was placed face down on a cover slip before being submerged in liquid nitrogen. The filter was then removed from the coverslip before being examined using a Nikon Eclipse T*i*2 inverted microscope.

#### 2.3.2. Structural Analysis of Aggregates

Aggregates were filtered (100 mL) onto 0.4 μm polycarbonate filters (Merck Millipore Ltd.) under gentle vacuum before being cut into 1 cm^2^ samples. The samples were then fixed and dehydrated, following the previously described protocol of Lee et al. [61]. Each sample was prepared for imaging following the protocol of Jamieson et al. [62]. The energy dispersive X-ray (EDX) spectroscopic analysis was conducted at 10 kV for 2000 s. 

#### 2.3.3. Extracellular DNA of Aggregates

Aggregates were filtered onto 0.4 μm polycarbonate filters (Merck Millipore Ltd.) under low vacuum. The filters were then stained with PicoGreen^TM^ (Invitrogen, Waltham, MA, USA) and examined using a Nikon Eclipse T*i*2 inverted fluorescence microscope.

### 2.4. Membrane Autopsy

The membrane modules obtained for this project were installed on the 19th of August 2010 and removed on the day of sampling, 1 September 2014. In total, four fouled SWRO membrane modules were provided by SA Water for an autopsy study: a membrane module from each stage of the SWRO unit (1st and 2nd stage), which had been in service for two and four years [62]. 

#### Membrane Autopsy TEP Quantification

The TEP present within the different sections of the SWRO membrane module were quantified. From the feed, the middle and end positions of the membrane three pieces were removed (1 cm × 10 cm) and stored in 0.2 µm filtered seawater at −20 °C, until analysis. The membrane samples were analysed as described by Balzano et al. [22] and Jamieson et al. [62].

### 2.5. DNA Extraction, Sequencing, and Bioinformatics

In order to identify the bacterial strains associated with TEP, nucleic acids were extracted from the water and aggregates, following the protocols described in Jamieson et al. [62]. The Ion Torrent platform sequence data was analysed using Mothur [63], following the previously described methods in Jamieson et al. [62]. The SILVA (version 132) and the Protist Ribosomal database (version 4.12.0) [64] was used to infer taxonomic affiliation of the OTUs using the UCLUST algorithm.

### 2.6. Data Analysis

The following statistical analyses were performed for both 16S and 18S rRNA sequencing data, unless noted. All data was transformed using Log+1 before undertaking Bray-Curtis similarity and Jaccard distance, in order to calculate similarity matrices between the prokaryote and eukaryote communities, respectively. The data were then analysed by principle coordinate analysis (PCoA) and similarity percentage (SIMPER) analysis using Primer7 (version 7.0.13). Differential abundance between two microorganism communities (intake water vs. SWRO feed tank water and intake water aggregates vs. SWRO feed tank water aggregates) were compared using the DESeq2 package (version 1.29.4) [65] using R (version 4.0.0). To identify the core, variable, and unique taxa among the water samples and aggregates, Venn diagrams were created with the online tool, access through https://bioinfogp.cnb.csic.es/tools/venny/ (accessed on 27 June 2021). The functional prediction of genes of the water and aggregate microbiota was acquired from web-based software Piphillin [66], based on the relative abundance of the OTU table (taxonomy was assigned with Silva database 132). Piphillin is a tool that assists with the prediction of metabolic profiles by mapping 16S sequences to known reference genomes: the KEGG pathways. The function prediction matrix was clustered and categorized utilising the Kyoto Encyclopaedia of Genes and Genomes (KEGG) orthologs (KOs) and pathways. Microsoft Excel was used to create the abundance graphs.

## 3. Results

### 3.1. Intake Seawater and SWRO Feed Tank Water Community Structure 

**Figure 2:**
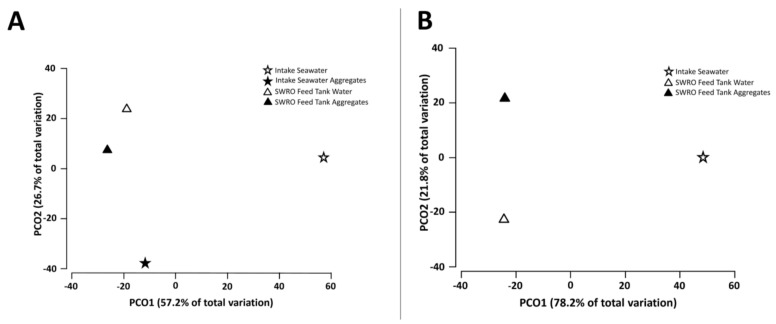
Principal coordinate analysis (PCoA) based on Bray-Curtis distance ordination, displaying the differences in the intake seawater and SWRO feed tank water in (**A**) the prokaryotic communities in the planktonic and aggregate-associated samples and (**B**) eukaryotic communities in the planktonic and aggregate-associated samples. The total variability is explained by the two PCoA axes, with the ordination of water samples, (**A**) explaining 83.9% of the attachment and pre-treatment variability observed in the samples and (**B**) 100% of the variability observed in the pre-treatment and attachment of the samples.

In this study, we investigated the planktonic and aggregate-associated communities present in the intake seawater, as well as in the SWRO feed tank water of the Penneshaw desalination system. Based on the 3106 bacteria, the OTUs identified in the water and/or aggregates after sequencing, dissimilarities in the prokaryotic community composition between the intake seawater, and SWRO feed tank water samples were identified using a PCoA. The separation along the principle coordinate PCO1 displays the dissimilarities in the prokaryotic community structure between the intake seawater and SWRO feed tank, reflecting the impact of the pre-treatment system within the plant. Whereas, along the principle coordinate PCO2, a lesser separation is evident between the water samples and aggregate communities, reflecting the community differences between the planktonic communities and those associated with the aggregates (Figure 2A). Similarly, differences in eukaryotic community structure were identified between the intake seawater and SWRO feed tank, using the 1208 eukaryote OTUs identified after sequencing (Figure 2B). Additional factors, such as the water pH and turbidity, potentially influence not only the prokaryotic and eukaryotic diversity within desalination plant but also the formation of the aggregates and their inhabitants.

### 3.2. Intake Seawater and SWRO Feed Tank Water Community Composition

**Figure 3:**
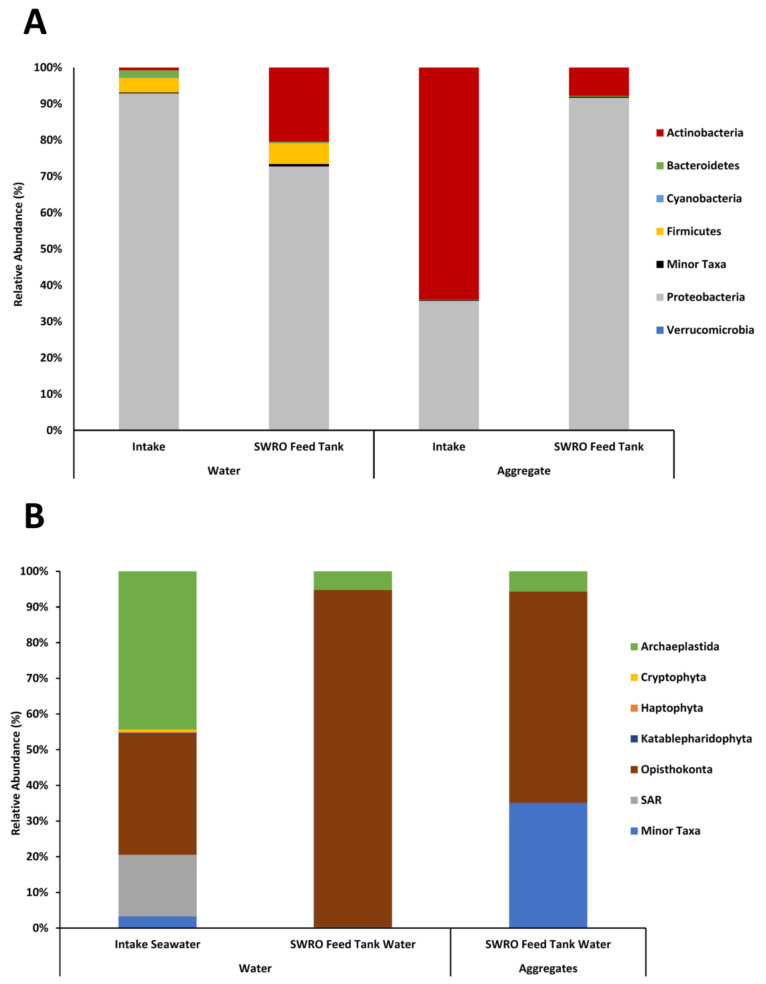
The relative abundance composition of the phylum taxonomy of the intake seawater and SWRO feed tank water samples (**A**) of the prokaryotic communities of the water and aggregate sample and (**B**) eukaryotic communities of the water and aggregate samples.

A total of 13 bacterial taxa were identified in the water samples: Acidobacteria, Actinobacteria, Bacteroidetes, Chloroflexi, Cyanobacteria, Firmicutes, Fusobacteria, Gemmatimonadetes, Marinimicrobia, Patescibacteria, Proteobacteria, and Synergistetes. The phyla Proteobacteria was the most dominant (82.8%), followed by Actinobacteria (10.6%), Firmicutes (4.9%), and Bacteroidetes (1%). Minor taxa consisted of the phylum Acidobacteria, Chloroflexi, Fusobacteria, Gemmatimonadetes, Marinimicrobia, Patescibacteria, and Synergistetes (Figure 3A). The phyla Cyanobacteria, Marinimicrobia, Acidobacteria, and Verrucomicrobia were only found in the intake seawater, while the phyla Chloroflexi and Gemmatimonadetes were only identified in the SWRO feed tank water. Pielou’s evenness values indicate that OTU abundances within the seawater samples were highly diverse but similar across samples (Table 1). SIMPER analysis determined a significant dissimilarity between the prokaryotic communities in the intake water, and the SWRO feed water was 82.57%. The dissimilarity was due to a significant increase in *Pseudomonas*, *Aeromonas*, *Streptococcus*, *Rahnella*, *Cedecea*, *Stenotrophomonas*, *Cutibactium*, and *Staphylococcus* in the SWRO feed tank water and significant decrease in *Effusibacillus* and *Pseudoalteromonas*.

Six eukaryotic taxa were identified in the water samples: Archaeplastida, Opisthokonta, Cryptophyta, Haptophyta, Katablepharidaceae, and SAR, in line with the revised Eukaryotic classification put forward by Adl et al. [67]. The supergroup of Opisthokonta was the most dominant (intake water 53.8%, SWRO feed tank water 96.10%), followed by Archaeplastida (intake water 43.87%, SWRO feed tank water 3.10%; Figure 3B). The groups Cryptophyta, Haptophyta, and Katablepharidaceae were only found in the intake seawater. Pielou’s evenness values indicate that OTU abundances within the seawater samples were highly diverse but similar across samples (Table 1). SIMPER analysis determined a significant dissimilarity between the eukaryotic communities in the intake seawater, and the SWRO feed tank water of 66.68%. This was due to a significant increase in the abundance of the two Opisthokonta classes of Sordariomycetes and Exobasidiomycetes. 

### 3.3. Aggregate Composition, and Size

**Figure 4:**
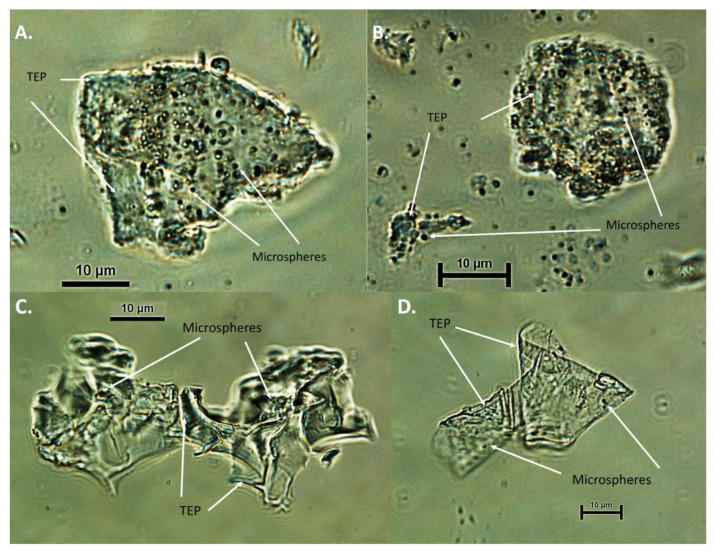
Microscopy of alcian blue stained aggregate samples, formed in the Penneshaw desalination plant. (**A**,**B**) Aggregates formed in the intake seawater and (**C**,**D**) aggregates formed in the SWRO feed tank water fluoresced cells (Figure 5A,B). The larger, brighter cells could be attributed to bacteria within the aggregates, with the smaller-sized particles, denoting a diffusion of the eDNA into the EPS surrounding the aggregates. The aggregate sample formed in the SWRO feed tank water displayed uniformity in the coverage of eDNA, with more bacteria cells visible in the aggregates. Similarly, there is also the smaller-sized particles evident, surrounding the bacteria cells (Figure 5C,D).

**Figure 5:**
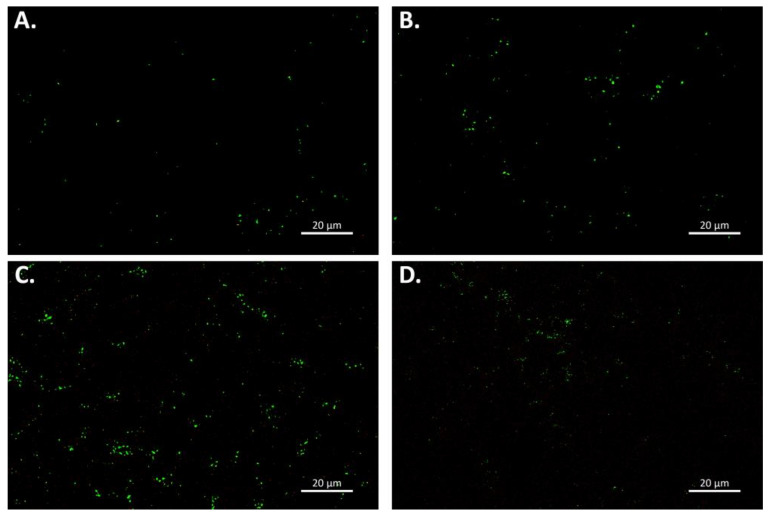
PicoGreen staining of extracellular DNA in the aggregates formed in Penneshaw SWRO desalination plant water. (**A**,**B**) Aggregates formed in the intake seawater. (**C**,**D**) Aggregates formed in SWRO feed tank water.

To observe the role of TEP in the formation of the aggregate, acidic alcian blue stain was applied. The aggregates, observed under ×40 microscopy, formed from the intake seawater, were of a viscous nature, in which the magnetic beads were apparent (Figure 4A,B). The alcian blue staining shows the presence of TEP particles in many of the aggregates; however, it is not formed only from TEP particles. The aggregates, formed in the SWRO feed tank water, are of a gelatinous nature, in which the magnetic beads can be observed. Alcian blue staining of the aggregate is apparent, and the complete aggregate is not stained (Figure 4C,D). PicoGeen was used to visualise the extracellular DNA (eDNA) in the aggregates. The staining of the aggregates, formed in the intake seawater, for eDNA, displayed two distinct sizes of fluoresced cells (Figure 5A,B). The larger, brighter cells could be attributed to bacteria within the aggregates, with the smaller-sized particles denoting a diffusion of the eDNA into the EPS, surrounding the aggregates. The aggregate sample, formed in the SWRO feed tank water, displays as uniformity in the coverage of eDNA, with more bacteria cells visible in the aggregates. Similarly, there are also smaller-sized particles evident, surrounding the bacteria cells (Figure 5C,D).

**Figure 6:**
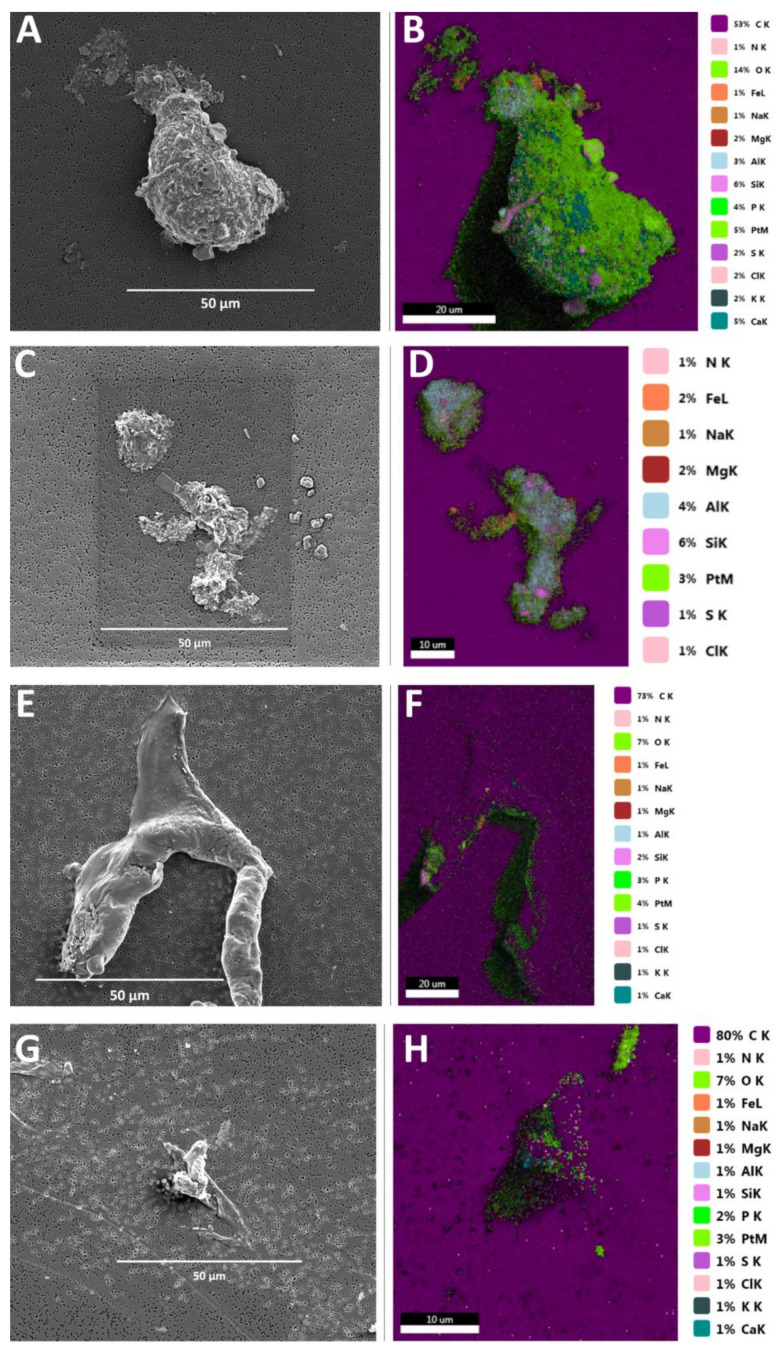
Scanning electron microscope images of an aggregates, created within the intake water (**A**,**C**) and corresponding energy dispersive X-ray (EDX) spectroscopic analysis (**B**,**D**). Aggregates formed within the RO feed tank water (**E**,**G**), alongside the EDX spectroscopic analysis of the protobiofilm (**F**,**H**).

Scanning electron microscopy (SEM) and energy-dispersive X-ray analysis (EDX) were applied to the aggregates to determine their structure and chemical composition. This technique gives an overall mapping of the aggregates by analysing near-surface elements and estimating their elemental proportion at different positions by moving the electron beam across the aggregates. The aggregates formed within the intake seawater were robust in structure, with a large amount of debris attached (Figure 6A,C). In contrast, those formed within the SWRO feed tank water presented a more viscous structure, with limited debris (Figure 6E,G). SEM-EDX analysis of the aggregates showed that their elemental composition was similar for the intake (Figure 6B,D) and SWRO feed tank water (Figure 6F,H). The presence of carbon, nitrogen, iron, sodium, magnesium, aluminium, silicon, sulphur, chlorine, potassium, chromium, nickel, and calcium was detected in the aggregates in varying concentrations (Table 2). The aggregates analysed showed that the chemical composition of the structures is complex and variable. The aggregate formed in the intake seawater contained relatively low abundances of carbon, in comparison to those formed in the SWRO feed tank water, which contained moderate amounts. Conversely, the intake seawater aggregates contained moderate amounts of oxygen, whereas those formed in the SWRO feed tank water displayed only low abundances. The elements chromium and nickel were only found to be present in the SWRO feed tank aggregate.

### 3.4. Aggregate-Associated Community Composition

Nine bacterial taxa were detected in the aggregates: Actinobacteria, Bacteroidetes, Cyanobacteria, Firmicutes, Fusobacteria, Patescribacteria, Proteobacteria, and Synergistetes. The phylum Proteobacteria dominated the community (63.7%), followed by Actinobacteria (35.9%), and the minor taxa consisted of the phylum Synergistetes (Figure 3A). The phyla Cyanobacteria and Synergistetes were only found in the intake aggregates, whereas phyla Patescibacteria and Fusobacteria were only found in the SWRO feed aggregates. Pielou’s evenness indicates that OTU abundances within the aggregates were highly diverse and equally distributed (Table 1). SIMPER analysis determined that the significant dissimilarity between the prokaryotic communities in the intake and SWRO feed tank water aggregates was 59.14%. This was due to a significant increase in the γ-proteobacteria genus Cutibactium, Delftia, Serratia, Rahnella, and Cedecea in the SWRO feed tank water. While a significant decrease in the γ-proteobacteria genera Pseudomonas, α-proteobacteria genera Altererythrobacter, and Actinobacteria genera Cornebacterium in the SWRO feed tank water also contributed to the dissimilarity. The functional prediction of genes of the water and aggregate bacteria was acquired from web-based software Piphillin. Several pathways, i.e., amino acid metabolism, carbohydrate metabolism, folding, sorting, and degradation, metabolism of cofactors and vitamins, biosynthesis of other secondary metabolites, glycan biosynthesis, and metabolism were identified to be significantly higher (*p* < 0.05) in the SWRO feed aggregates, compared to the intake seawater aggregates (Table 2).

Three eukaryotic taxa were detected in the aggregates formed in the SWRO feed tank water: Archaeplastida, Opisthokonta, and SAR. The supergroup of Opisthokonta dominated the community (92.6%), followed by Archaeplastida (5.8%) (Figure 3B). Pielou’s evenness indicates that OTU abundances within the aggregate sample were highly diverse (Table 1). From the intake seawater aggregates, the no 18S rRNA region was amplified, due to low levels of rDNA extracted.

### 3.5. SWRO Membrane Modules Autopsy

Overall, there was a significantly higher concentration of TEP (*p* < 0.05) on the membrane module in service for 4 years, compared to the 2-years membrane module. The TEP concentration of the 2-year membrane modules at stage 1 (783.77 ± 58.19 µG·Xg·L^−1^·m^−2^) was significantly higher than that found at stage 2 (459.01 ± 48.34 µg·Xg·L^−1^·m^−2^). Whereas, for the 4-years membrane modules, the TEP concentration between the stage 1 (1055.9 ± 46.93 µg·Xg·L^−1^·m^−2^) was comparable (*p* > 0.05) to that of stage 2 (1013.96 ± 27.67 µg·Xg·L^−1^·m^−2^).

**Figure 7:**
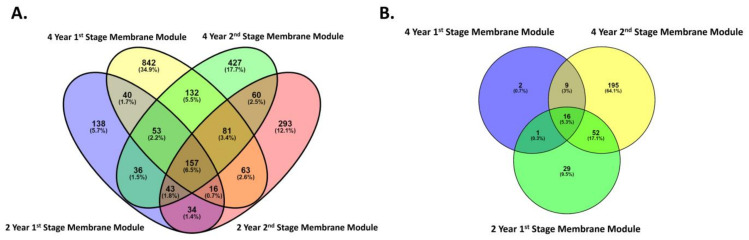
Venn diagram displaying the overlap between the (**A**) prokaryotic communities in the 2- and 4-membranes in the 1st and 2nd stage positions and (**B**) eukaryotic communities in the 2- and 4-membranes modules in the 1st and 2nd stage positions. Core OTUs, identified in all sampling sites; variable OTUs, identified in two or more sites but not all; unique OTUs, identified in only one site.

Venn diagrams were used to identify the core OTUs between the stage 1 and 2 membrane modules after 2 and 4 years of service. These organisms are considered essential to the function of their communities, thus reflecting a “healthy” population and influence or effect of any impediment [68]. Three different groups were identified: core OTUs (identified in all sampling sites), variable OTUs (identified in multiple sites but not all), and unique OTUs (identified in only one site). There were 2415 prokaryotic OTUs obtained from the SWRO membranes of stages 1 and 2 after 2- and 4-years operation, of which 70.4% were unique OTUs, 23.1% were variable OTUs, and 6.5% were core OTUs (Figure 7A). The core OTUs included twelve classes within eight phyla. The classes of core OTU’s were found to be Acidimicrobiia, Actinobacteria, α-proteobacteria, Anaerolineae, Babeliae, Bacteroidia, ε-proteobacteria, γ-proteobacteria, Gracilibacteria, Oxyphotobacteria, Phycisphaerae, and Thermoleophilia. 

Of the 304 eukaryote OTUs obtained from the 2-year, as well as the 4-year, Stage 1 and 2 membranes, 16 (5.3%) OTUs were considered core, 62 (20.4%) were considered variable OTUs, and 226 (74.3%) OTUs were considered unique (Figure 7B). The OTUs were core consisted of five classes within five phyla. The core OTUs are dominated by the class of Chloropicophyceae, Dinophyceae, Peronosporea, Sordariomycetes, and Trebouxiophyceae. 

### 3.6. Aggregate Fouling Potential

**Figure 8:**
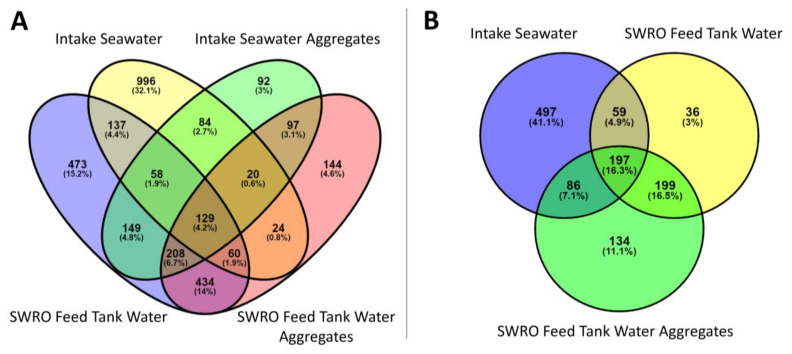
Venn diagram displaying the overlap between the (**A**) prokaryotic and (**B**) the eukaryotic communities in the water and protobiofilms. Core OTUs, identified in all sampling sites; variable OTUs, identified in two or more sites but not all; unique OTUs, identified in only one site.

To further the understanding of the differences between water and aggregate communities, core OTUs were identified using Venn diagrams. Three different groups were identified: core OTUs (identified in all sampling sites), variable OTUs (identified in multiple sites but not all), and unique OTUs (identified in only one site). Of the 3106 bacteria OTUs identified in the water and/or the aggregates, 1705 OTUs (55%) are considered unique, 1331 OTUs (41%) are considered variable, and 129 OTUs (4%) are considered core (Figure 8A). The core OTUs consisted of seven classes, within four phyla. The core OTU classes were found to be Actinobacteria, Bacilli, Gracilibacteria, α-proteobacteria, β-proteobacteria, ε-proteobacteria, and γ-proteobacteria. Of the 1208 eukaryote OTUs identified in the water and/or the aggregates: 667 OTUs (55%) are considered unique, 344 (29%) are considered variable, and 197 (16%) are considered core (Figure 8B). The core OTUs are dominated by the classes of Ascomycota, Basidiomycota, and Chlorophyta.

**Figure 9:**
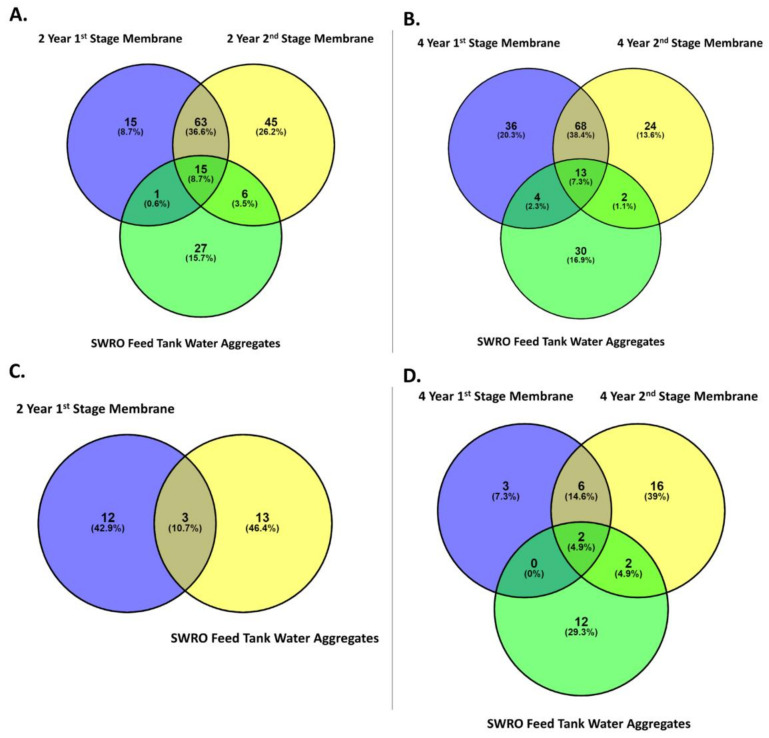
Venn diagram displaying the overlap between the communities of the aggregate and the 1st and 2nd stage membranes. (**A**) Prokaryotic communities in the SWRO feed tank water aggregates, and the 1st stage and 2nd stage membrane after two years’ service and (**B**) the prokaryotic communities in the SWRO feed tank water aggregates and 1st stage and 2nd stage membranes after four years of service. (**C**) The eukaryotic communities in the SWRO feed tank water aggregates and 1st stage membrane after two years’ service and (**D**) eukarotic communities of the SWRO feed tank water aggregates and 1st stage and 2nd stage membranes after four years of service. Core OTUs, identified in all sampling sites; variable OTUs, identified in two or more sites but not all; unique OTUs, identified in only one site.

In order to assess the fouling potential of the aggregate communities, these were compared to the communities found in fouled membrane modules, extracted from the Penneshaw desalination plant [62] using Venn diagrams. Here, OTUs were compared at family level. Of the 239 prokaryote OTUs found in the aggregates and the SWRO membrane module after 2-years’ service, 87 OTUs (51%) are considered unique, 78 (40%) are considered to be variable, and 15 (9%) are considered to be core (Figure 9A). The OTUs considered to be essential were dominated by the families of the Proteobacteria phylum, including *Sphingomonadaceae*, *Rhodobacteraceae*, *Parvularculaceae*, *Legionellaceae*, *Parvibaculaceae*, *Xanthobacteraceae*, SAR116 clade, *Burkholderiaceae*, and *Pseudomonadaceae*, as well as *Propionibacteriaceae* from the Actinobacteria phylum and *Flavobacteriaceae* from the Bacteroidetes phylum. From the aggregates and 4-year-old SWRO membrane modules, 213 prokaryote OTUs were analysed, of which, 90 OTUs (51%) are considered unique, 74 (42%) are considered variable, and 13 (7%) are considered core (Figure 9B). The essential OTUs are dominated by the families of the phylum Proteobacteria: *Rhodobacteraceae*, *Sphingomonadaceae*, *Parvularculaceae*, *Parvibaculaceae*, *Legionellaceae*, *Xanthobacteraceae*, PS1 clade, *Burkholderiaceae*, PS1 clade, and SAR116 clade, as well as *Propionibacteriaceae* from the phylum Actinobacteria and *Flavobacteriaceae* from the Bacteroidetes phylum.

The aggregates and the 1st stage 2-year-old SWRO membrane module consisted of 28 eukaryote OTUs at the class level (Figure 9C), of which 25 OTUs (89%) are considered unique OTUs, and 3 OTUs (11%) are considered to be core OTUs. These were dominated by the classes of Sordariomycetes and Dothideomycetes, of the super group Opisthokonta, as well as the Stramenopiles supergroup Dinophyceae. Of the 37 eukaryote OTUs found in aggregates and 4-year-old SWRO membrane modules, 23 OTUs (76%) are considered unique, 9 (19%) are considered variable, and 2 (5%) are considered core (Figure 9D). The essential OTUs are dominated by the classes of Sordariomycetes and Dinophyceae.

## 4. Discussion

Within water treatment plants, it is widely recognised that pre-treatment systems are essential for the efficient production of potable water. This novel study looks at the microbial composition and biofouling potential of aggregates formed within a SWRO desalination plant. A study conducted by Balzano et al. [22] established that the use of pre-treatment, especially multimedia filtration, within the desalination system had the ability to reduce the microbial biomass by one order of magnitude, thereby affecting change within the planktonic prokaryotic and eukaryotic community composition within the desalination plant. However, pre-treatment systems also create niche environments, thereby producing conditions that are favourable for the development of aggregates and proliferation of organisms. 

### 4.1. Prokaryotic Communities in Water

Seasonal fluctuations of nutrients, microorganisms, and phytoplankton have been previously described within the Penneshaw SWRO desalination plant [22,69]. This results in a highly diverse, yet unique, microbial community within the intake seawater and SWRO feed tank water. The composition of the prokaryotic community observed in the intake seawater and in the SWRO feed tank was consistent with that previously observed in SWRO desalination plants globally [52,53,54,70]. Here, it was observed that Proteobacteria and Actinobacteria were the dominant phyla within the intake seawater and the SWRO feed tank water. Proteobacteria and Actinobacteria, along with Bacteroidetes, Cyanobacteria, and Verrucomicribia, are amongst the most abundant phyla within the marine environments [71,72,73]. Verrucomicribia, in particular, is a polymer-degrading bacterium, commonly associated to marine POM.

Within water treatment systems, the classes of Proteobacteria are often the most dominant organism, identified not only within the intake water but also in fouled membrane modules [74]. Proteobacteria classes of α- and γ-proteobacteria, as well as the class of Actinobactiera, are commonly found within the intake water of desalination plant [52,53]. Furthermore, both α- and γ-proteobacteria abundance can increase after pre-treatment [52,53], and could be a result of the development of organic compound layers within some of the components of the pre-treatment systems, such as cartridge filters [49,51]. In general, α-proteobacteria are often considered to be the primary colonizers within biofilms [42,75,76], whereas β-proteobacteria are more commonly associated with fouled membrane modules within desalination plants, as they have a key role in mature biofilm development [76,77].

### 4.2. Eukaryotic Communities in Water

The eukaryotic communities identified within the intake seawater and SWRO feed waters are similar to those present in marine ecosystems [52,78]. Both phototrophic and heterotrophic eukaryotes have an important role within the marine environment, especially in primary production, respiration, and the microbial loop [79]. The eukaryotic communities observed in the water samples are also consistent with those previously observed in SWRO desalination plants [80]. For example, fungi were present in both the intake seawater and SWRO feed tank water; however, it is the classes of Sordariomycetes and Exobasidiomycetes that drive the diversity between the water samples. While the role and impact of fungi within biofilms of water treatment systems is in the initial stages of research, the formation of biofilms by fungi, especially those developed by *Aspergillus fumigatus* and *Candida albicans*, are well-documented [81,82,83,84,85]. 

The green algae family members of the class Chlorophyta often dominate the picoplankton biomass and have an important role in the marine food web [86]. However, they are known to inhabit a wide variety of marine ecosystem; although, their distribution is influenced by their ability to adapt to environmental conditions [87]. *Ostreococcus* was present in the water samples, as well as in the SWRO feed tank aggregates. The genera of *Ostreococcus* is within the pico size fraction of eukaryotes (<2–3 µm diameter) and a unicellular, non-flagellated green alga [88]. Due to a large surface area to volume ratio, *Ostreococcus* is known for its rapid growth in oligotrophic environments [88,89]. In addition, *Ostreococcus* has been shown to thrive under low irradiances [89], as encountered within the SWRO system. The *Bathycoccus* genera was present within the intake seawater, as well as the SWRO feed tank water aggregate. The *Bathycoccus* genera is a widespread oceanic green alga [87], which ranges in size from 1–2 µm, the cells have no flagella but are covered in a spider web pattern of scales [90]. The relatively small size of the *Ostreococcus* and *Bathycoccus* (<2 µm) would allow for them to pass freely through the cartridge filters (pore size 15 and 5 µm) within the Penneshaw desalination plant. Their ability to adapt to nutrient gradients within oligotrophic environments would also be advantageous for their survival within the desalination system [88,91,92]. 

### 4.3. Aggregate Communities

A novel aspect of this study is the examination of the aggregate-associated organisms within the formed microenvironments. Due to the niche environments created within the Penneshaw SWRO desalination plant, the attachment of organisms is a selective process reflected in the decreased diversity of the inhabitants. The presence of Cyanobacteria only within the intake aggregates is not unexpected, as they are known as oxygenic photosynthetic prokaryotes [93], which requires the use of light to generate CO_2_ from water [94]. Identified within the RO feed tank aggregates, Patescribacteria have the ability to succeed in oligotrophic environments. In addition to their ultra-small cell size, this would enable them to pass through the pre-treatment structures and flourish within the water treatment system [95]. On the other hand, Fusobacteria observed in both the intake seawater and SWRO feed tank water possess a tapered rod shape. This would allow them to enter the SWRO feed tank as they would readily fit through the different pore sizes of the cartridge filter [96]. In addition, Fusobacteria are known to have the ability to co-aggregate with many bacteria [97]. The colonisation of aggregates in any environment is multifaceted and relies heavily upon numerous factors, including the microorganism’s motility, ability to attach or detach, growth, mortality, the dynamics of the environment, organism interactions, and communication [98]. The nutrient richness of the aggregates contributes to its colonization by microorganism communities [99]. 

The colonisation of the aggregates with Burkholderiales and Sphingomonadales microbes is not surprising, as they are often identified within aqueous environments, as well as being associated with particle attachment [100,101,102,103]. Of particular note is the common association that these organisms have with biofilms and biofouling [42,76,104]. For example, β-proteobacteria have long been associated with biofilms, especially as a class that contains organisms that can pioneer biofilm formation. The success of the betaproteobacteria has been attributed to the ability of its cells to co-aggregate [105]. Burkholderiales have also been categorised as second colonisers of biofilms, preferring a pre-developed biofilm to adhere and grow upon [106]. Finally, Sphingomonadales are known to colonize aggregates, where they breakdown the polymer-rich substrates and later release them into the surrounding environment [107]. Within the SWRO feed tank, the broken-down aggregate substrates could serve as hot spots to promote biofilm growth in SWRO membrane modules [108,109]. While, in our study, we see a reduction in the colonization of aggregates by Burkholderiaceae, Janthinobacterium, and Sphingomonadales this would indicate that they are not reliant upon the colonization of aggregates to initiate the formation of biofilms that they are known for [42,110,111].

The taxonomic diversity of the eukaryotic organisms within the SWRO feed tank water and SWRO feed tank aggregates was very similar, mainly due to the abundance of planktonic and aggregate-bound Ascomycota and Basidiomycota organisms. The classes of Ascomycota and Basidiomycota form the subkingdom of Dikarya, which is principally made of fungi and often observed in marine environments [112,113,114,115]. The adaption of fungi to life in anaerobic and partially anaerobic environments, through cellular and genomic adaptions, allows them to flourish in any environment [116]. The presence of Fungi (92.51%) within the aggregates is not unusual, as fungi are commonly identified in the marine environments [117,118,119,120]. The diversity of fungi allows for these ubiquitous organisms to not only survive in marine and freshwater environments but to also perform key roles in the biogeochemical cycling and production of secondary metabolites [121]. Bochdansky et al. [122] determined that, within the marine snow particles, the contribution of fungal cells was similar to that of the prokaryotic cells, and they have been known to dominate cells counts, compared with eukaryotic cells. This suggests that within aggregates, fungi have a saprophytic or symbiotic lifestyle that relies on other prokaryotic cells.

The Opisthokonta is a large supergroup of eukaryotes, including metazoans, fungi, choanoflagellates, amoeboids, and sporozoan protists. These organisms are phagotrophic or osmotrophic. The nutrient-rich substrate of the formed aggregates potentially provides the optimum environment for the osmotropic lifestyle of these eukaryotic organisms [122]. 

### 4.4. Fouling Potential of Aggregates

SEM-EDX analysis was applied to the aggregates of the intake water and SWRO feed tank water. Similar chemical elements were found in fouled cartridge filters and SWRO membrane modules from within a commercial desalination plant [51], suggesting that the cartridge filters may trap some of the aggregates during the pre-treatment process. This trapping would create favourable conditions for microorganisms to flourish. However, due to the pressure within the system, the aggregates, which are trapped in the cartridge filter, could breakdown and pass through the pores, allowing the particles to hypothetically coagulate further downstream. The formation of TEP within environments is through the coagulation of dissolved organic matter [123]. As a consequence, the nature of TEP is highly viscous and has been reported to be 2–4 magnitudes higher than any other particle [27,124]. Thereby, ensuring a role in the aggregation/sedimentation process within the marine environment [123]. The polymer network that forms the TEP particle is negatively charged, thus absorbing surrounding organic molecules and trace metal [124]. Subsequently, the three-dimensional structure of TEP particles results in a large surface area, providing an environment with an abundance of nutrients [124]. Previous research has shown that, within freshwater and seawater, 0.5–25% of bacteria are attached to TEP particles [125,126]. 

The aggregate-associated communities were compared to those found in SWRO fouling, in order to assess the fouling potential of the aggregates within a desalination plant, with the aggregate-associated communities contributing to 10.7% of the communities identified in the 1st stage membrane module after two years of operation. A total of 4.9% of organisms were found to be consistent with the communities found in the 1st and 2nd stage membrane modules after four years of operation. The core OTUs are consistent with those identified in fouled SWRO membrane modules but also are known to form biofilms. Many of the core OTUs are ubiquitous in water treatment and distribution systems, as they have the ability to survive extreme conditions [127]. The essential OTUs, especially Sphingomonadaceae, Rhodobacteraceae, Legionellaceae, Burkholderiaceae, and Pseudomonadaceae, are commonly associated with biofilms and/or biofouling [42,128,129,130]. *Sphingomonas* has been identified as having a unique role in the fouling of SWRO membrane modules, especially in the formation of the initial biofilms [42]. They have also been recognised for their ability to survive high concentration of chlorine, which is directly linked to the production of EPS [131,132]. The Rhodobacteriaceae family are abundant within the marine environments and are often found to the be the primary colonisers within biofilms on submerged surfaces, as well as water treatment systems [128,133]. Rhodobacteria are known to contain gene transfer agents (GTA) a particle, which allows the transfer of fragments of genome DNA to be transferred to other cells [134]. While the focus of HGT is on the survival of cells, this is not the case with GTA, as it is not selective of the fragments transferred [134]. Allowing for the potential to not only disseminate virulence and antimicrobial resistance genes but also to force the evolution of bacteria [134]. Biofilm development in species within the Burkhoderiaceae family has been found to be positively correlated to the quantity of eDNA from living cells [135]. eDNA is essential for the attachment of cells, as well as during the development of the biofilm [136,137,138,139]. 

### 4.5. Future Considerations

The ability of particles to come together within a SWRO desalination plant after pre-treatment would suggest that the current methods of removal are both inadequate and ineffective. The conventional pre-treatment system within the Penneshaw SWRO desalination is limited; however, the adaption of novel treatments alongside the conventional pre-treatment system enhances the quality and quantity of potable water produced. Vertical wells (subsurface intake systems) have been successful in effectively improving the quality of the intake water of various desalination plants worldwide. The transfer aquifer reduces the fouling and biofouling constitutions before the seawater enters the well [140,141]. Another novel pre-treatment method that has success in reducing the biofouling potential of the water within the desalination system is that of the granular activated carbon (GAC) biofilters. Studies have shown that GAC biofilters were more effective in the removal of low molecular weight organics in the system than microfilters and ultra-filtration membrane modules, as well as reducing fouling precursors, such as TEP and assimilable organic carbon [142]. Coagulation is another novel method that is providing promising results in reducing the fouling potential of organisms. The addition of liquid ferrate, even at low levels, was effective in the reduction of fouling precursors, as well as the reduction of algal and bacteria cells within the feed seawater [143,144], thereby reducing the prospect of rapid fouling/biofouling in the SWRO membrane modules.

## 5. Conclusions

This study is the first to investigate the formation of aggregates within a SWRO desalination plant, examine the microbial community of the aggregates, and investigate the role they may have on membrane fouling. The prevalence of polysaccharide precursors within desalination plants has been established, as has the colonization of aggregates within the water column. Even though the water within a desalination plant undergoes multiple pre-treatment steps, the pressure driven SWRO system creates the perfect environment for the formation and inhabitation of aggregates. The pre-treatment systems removes larger particles, flocculation, and microorganisms, yet the smaller fragments have the ability to come together to form aggregates further in the system. These aggregates are a hot spot for nutrients and enable the formation of niche communities within. Evidence suggests that, within these hot spots, the transfer of genes allows the attached microorganisms a competitive edge to survive in such an oligotrophic environment, where they are able to persist in the developed biofilms. Future work should focus on whether the removal of aggregates from the system reduces biofouling within SWRO desalination plants. The introduction of smaller pore size within the cartridge filters to remove the <5 µm organisms or introduction of another pre-treatment system, such as coagulation prior to the SWRO membrane modules (i.e., after the feed tank), may help to further reduce the biofouling precursors reaching the SWRO membrane modules. 

## Figures and Tables

**Table 1:** Pielou’s evenness values of the prokaryotic and eukaryotic organisms in the water, aggregates, and biofouled membranes analysed in the present study.

Sampling Site	Prokaryotes Pielou’s Evenness	Eukaryotes Pielou’s Evenness
Intake Seawater	0.96	0.97
Intake Seawater Aggregates	0.96	
SWRO Feed Tank Water	0.96	0.96
SWRO Feed Tank Water Aggregates	0.96	0.97
2-years 1st Stage SWRO membrane	0.64	0.93
2-years 2nd Stage SWRO membrane	0.58	
4-years 1st Stage SWRO membrane	0.51	0.96
4-years 2nd Stage SWRO membrane	0.60	0.93

**Table 2:** The elemental composition and proposed biological and chemical components of the aggregates, formed within the Penneshaw SWRO desalination plant intake seawater and SWRO feed tank water, analysed in the present study.

	Intake Seawater Aggregate 1	Intake Seawater Aggregate 2	SWRO Feed Tank Aggregate 1	SWRO Feed Tank Aggregate 2
Chemical Elements	C, N, O, Fe, Mg, Al, Si, S, Cl, Ca	C, N, O, Fe, Mg, Al, Si, Cl, K, Ca	C, N, O, Fe, Na, Al, Ca, K, Cl, S	C, N, O, Fe, Na, Al, Si, Cl, K, Ca, Cr, Ni
Proposed Biological and Chemical Components	Shell/Bone Diatom Aluminosilicate Iron Oxide Calcium Silicate Polysaccharide	Polysaccharide Aluminosilicate Salt–CaCl_2_	Salt–KCl, NaCl Sulfate Iron Oxide Calcium Polysaccharide	Stainless steel Salt–NaCl, KCl, CaCl_2_ Tentative–CaSO_4_ Iron Oxide Polysaccharide Silica
